# An Efficient Algorithm for Small Livestock Object Detection in Unmanned Aerial Vehicle Imagery

**DOI:** 10.3390/ani15121794

**Published:** 2025-06-18

**Authors:** Wenbo Chen, Dongliang Wang, Xiaowei Xie

**Affiliations:** 1 Key Laboratory of Land Surface Pattern and Simulation, Institute of Sciences and Natural Resources Research, Chinese Academy of Sciences, Beijing 100101, China; 2022110416@ecut.edu.cn; 2 School of Surveying and Geoinformation Engineering, East China University of Technology, Nanchang 330013, China; xwxie@ecut.edu.cn; 3 Jiangxi Key Laboratory of Watershed Ecological Process and Information (Platform No. 2023SSY01051), East China University of Technology, Nanchang 330013, China; 4 Key Laboratory of Mine Environmental Monitoring and Improving Around Poyang Lake of Ministry of Natural Resources, East China University of Technology, Nanchang 330013, China

**Keywords:** unmanned aerial vehicle (UAV), deep learning, object detection, livestock population surveys

## Abstract

Livestock population surveys are crucial for grassland management tasks such as health and epidemic prevention, grazing prohibition, rest grazing, and forage–livestock balance assessment. These tasks are integral to the modernization and upgrading of the livestock industry and the sustainable development of grasslands. Unmanned aerial vehicles (UAVs) provide significant advantages in flexibility and maneuverability, making them ideal for livestock population surveys. However, grazing livestock in UAV images often appear small and densely packed, leading to identification errors. To address this challenge, we propose an efficient Livestock Network (LSNET) algorithm, a novel YOLOv7-based network. Our approach incorporates a low-level prediction head (P2) to detect small objects from shallow feature maps, while removing a deep-level prediction head (P5) to mitigate the effects of excessive down-sampling. To capture high-level semantic features, we introduce the Large Kernel Attentions Spatial Pyramid Pooling (LKASPP) module. In addition, we replaced the original CIoU with the WIoU v3 loss function. Furthermore, we developed a dataset of grazing livestock for deep learning using UAV images from the Prairie Chenbarhu Banner in Hulunbuir, Inner Mongolia. Our results demonstrate that the proposed module significantly improves the detection accuracy for small livestock objects, with the mean Average Precision (mAP) increasing by 1.47% compared to YOLOv7. Thus, this work offers a novel and practical solution for livestock detection in expansive farms. It overcomes the limitations of existing methods and contributes to more effective livestock management and advancements in agricultural technology.

## Simple Summary

Precise livestock detection is crucial for livestock population surveys. However, livestock in unmanned aerial vehicle imagery are often small and densely distributed, leading to suboptimal detection performance. To address this issue, we propose a novel small-object livestock detection method. Experimental results demonstrate that proposed method significantly enhances the accuracy of livestock detection while achieving a lightweight model, providing an effective technical solution for livestock population surveys.

## 1. Introduction

China is a major nation in grassland animal husbandry, which is a crucial component of agricultural production [1]. Accurate and timely livestock data are vital for grassland management tasks, including sanitation, epidemic prevention, grazing prohibition, rest grazing, and forage–livestock balance assessment [2].

Traditional methods for monitoring animal aspects, such as population size and structure, predominantly rely on ground-based surveys. These methods facilitate close observation of animal behavior and population, as well as the collection of animal traces and forage samples. However, they are labor-intensive, time-consuming, costly, prone to producing repetitive results, and may be constrained by terrain [3]. Satellite-based monitoring typically utilizes very high-resolution (0.31–1 m panchromatic resolution) satellite images to count animals. These methods offer extensive coverage, frequent revisit intervals, and do not disturb the animals. However, they are effective only for recognizing large individual animals (greater than 0.6 m), such as wildebeests and zebras [4]. In contrast, Unmanned Aerial Vehicles (UAVs) are agile, cost-effective, quiet, less restricted by natural terrain, and can maintain a safe distance from animals, minimizing disturbance. Consequently, UAVs equipped with high-resolution cameras have been used to quickly and accurately capture small-sized animals and juvenile individuals, and subsequently count their numbers [5].

Previous surveys primarily relied on manual observation and counting from large volumes of images. This method can be tedious, costly, and prone to errors, leading to lower detection rates and miscalculations. As a result, researchers have proposed various automatic or semi-automatic object detection algorithms to improve survey efficiency. Pixel-based classification methods, including supervised and unsupervised classification, as well as threshold setting, have been developed. For example, Gonzalez et al. [6] created an algorithm to automatically count and track koalas, deer, and kangaroos in UAV RGB and thermal imaging videos using threshold segmentation and template matching techniques. Thresholding and unsupervised classification algorithms were also developed to detect and count chickens in agricultural fields using thermal imagery, reducing animal mortality caused by agricultural machinery. Additionally, these algorithms have been used to identify black-faced spoonbills (Platalea minor) in UAV RGB imagery, aiding the annual international black-faced spoonbill census [7]. LaRue et al. [8] aimed to expedite detection by automating the identification of polar bears through supervised spectral classification and image differencing. Xue et al. [9] developed a novel semi-supervised, object-based method that combines a wavelet algorithm and fuzzy neural network to detect and count wildebeests and zebras from a single GeoEye-1 panchromatic image in open savannas. Compared with traditional thresholding techniques, this method produced a higher accuracy index and fewer omission errors. With the availability of higher-resolution images, researchers have proposed various algorithms combining machine learning to extract more complex features. For instance, using rotation-invariant object descriptors with machine learning algorithms, Torney et al. [10] implemented an algorithm to detect and count wildebeest from aerial images taken in Serengeti National Park. While the algorithm was more accurate than manual counts, it showed a higher per-image error rate, with a detection precision of 74.15%. Rey et al. [11] proposed a data-driven machine-learning system for the semi-automatic detection of large mammals, including common elands (*Taurotragus oryx*), greater kudus (*Tragelaphus strepsiceros*), and gemsboks (*Oryx gazella*) in the Savanna ecosystem, using 6500 RGB UAV images. The system achieved a recall rate of 75% for a precision of 10%. Christiansen et al. [12] proposed a thermal feature extraction algorithm using the discrete cosine transform (DCT) to parameterize thermal signatures. They employed a k-nearest-neighbor (kNN) classifier to automatically distinguish wild rabbits and chickens from non-animal objects, achieving classification accuracy of 93.3% at altitudes ranging from 3 to 10 m. Although these methods exhibit good recognition accuracy for small-scale datasets and simple scenes, they perform poorly in complex scenarios involving lighting variations, occlusions, and diverse poses. They also struggle with large-scale, multi-class, and highly abstract recognition tasks, and exhibit weak generalization ability.

In recent years, deep learning technologies have developed rapidly and achieved significant results in image processing. Compared to traditional visual recognition techniques, which only extract shallow image features, deep learning methods can automatically learn richer semantic information and higher-level image features from vast amounts of data, allowing them to discern differences between various types of objects. Convolutional neural networks (CNNs), a class of advanced deep learning models with multiple hidden layers and deeper architectures, can extract specific information, such as the posture and body length of animals in higher-resolution images. Currently, CNN-based object recognition algorithms can be broadly categorized into two-stage and single-stage object detection algorithms. Two-stage object detection algorithms consist of two primary phases: the first phase involves generating candidate regions, also known as region proposals, and the second phase involves classifying these candidate regions and performing precise localization. Notable examples in this category include R-CNN, Faster R-CNN, and Mask R-CNN. In contrast, single-stage object detection algorithms directly extract features to generate regions of interest, classify each region, and produce bounding boxes. Notable models in this category include the You Only Look Once (YOLO) series and Single Shot MultiBox Detector (SSD). The YOLO series demonstrated high performance in locating and classifying the object of interest within an image by drawing bounding boxes and class probabilities. Consequently, researchers have adopted these methods for detecting animal objects in UAV remote-sensing images. Wuthrich [13] assessed the accuracy of Faster R-CNN, RetinaNet, and YOLOv9 for sheep counting in complex environments. They found that UAVs and object detection algorithms have significant potential for practical applications in improving the efficiency of livestock monitoring. Based on You Only Look Once v5, Deng et al. [14] developed a real-time detection system to count sheep, aiming to enhance counting accuracy. To address the challenges of missed and false detections of sheep of varying sizes in complex backgrounds, Cao et al. [15] proposed an improved YOLOv5x model based on the attention mechanism and the DeepSort algorithm, achieving a 5% error rate in dynamic sheep counting. Yu et al. [16] presented a method for monitoring dairy cow feeding behavior using the DenseResNet-You Only Look Once (DRN-YOLO) deep learning approach, achieving a 1.70% improvement in precision compared to the YOLOv4 model. To achieve real-time detection of small-target individual sheep from a high-altitude UAV perspective, Zhi et al. [17] developed a high-precision small-target detection model, CSD-YOLOv8s. Compared to the YOLOv8s model, precision improved from 93.0% to 95.2%, showcasing a significant enhancement.

Despite the significant achievements of deep learning in UAV-based animal detection, several challenges remain in detecting grazing livestock in grasslands. The detection of grazing livestock in UAV images differs significantly from that in ground-based images and other remote sensing detection tasks, leading to three main challenges. First, due to the varying angles of view of the drone camera, the target will appear differently. For instance, when the camera is at a vertical angle, there is generally a certain distance between dense objects, making it difficult for adjacent objects to overlap. However, when viewed from an oblique angle, dense targets appear to overlap. Second, to avoid disturbing livestock and to effectively cover a wide area, the UAV’s flight altitude is typically high. As a result, the objects in the captured images vary greatly in scale, with buildings, mountains, and livestock often appearing in the same image. Livestock occupies a very small portion of the image, making it challenging to extract useful and distinctive features for detection. Third, densely packed object areas in UAV images contain many identical objects, increasing the likelihood of false detections. Furthermore, noise in the background of UAV images can obscure or weaken the detection of objects.

1. We established a grazing livestock dataset based on UAV imagery data from the Prairie Chenbarhu Banner in Hulunbuir. This dataset serves as the foundation for our proposed LSNET algorithm, which enhances livestock target detection by adding a low-level prediction head (P2) to improve feature extraction performance for small livestock targets and removing the deep prediction head (P5) to reduce the impact of excessive down-sampling.
2. To further improve detection accuracy, the SPPCSPC module of YOLOv7 was enhanced to extract high-level semantic features by extending the receptive field and employing the Large Kernel Attention Mechanism. Moreover, incorporating WIoU v3 into the bounding box regression loss function.
3. Evaluated on the Hulunbuir grassland livestock dataset, the proposed LSNET algorithm achieves a mean Average Precision (mAP) of 93.33%, representing a 1.47% improvement over the traditional YOLOv7 algorithm, highlighting its superior performance in livestock detection tasks.

## 2. Materials and Methods

### 2.1. Experimental Data

**Figure 1:**
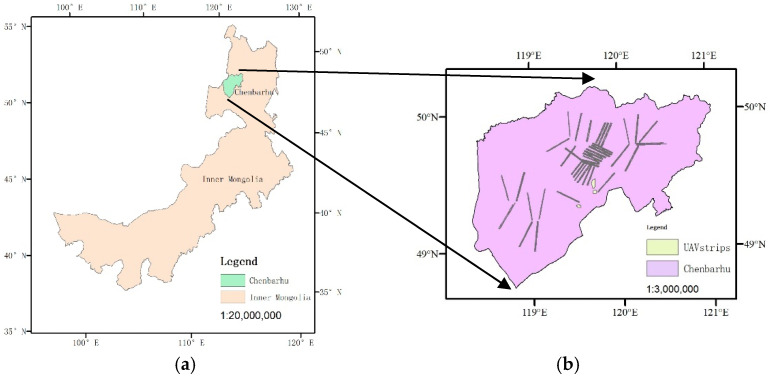
Study area position (**a**), flight path (**b**).

The experimental data were collected in the Prairie Chenbarhu Banner (48°48′ N–50°12′ N and 118°22′ E–121°02′ E) in Hulunbuir City, Inner Mongolia, China (Figure 1). It is one of the key animal husbandry production regions in Hulunbuir, with the predominant livestock types being cattle, sheep, and horses. From 13 July to 26 July 2023, a total of 45 UAV flights, conducted at vertical viewing angles, were carried out over the northwest part of the Prairie Chenbarhu Banner. The survey followed a systematic sampling method with a sampling intensity of 2.15%. The flight altitude was set at 300 m above ground, and the image spatial resolution ranged from 4 to 7 cm. A total of 30 flight strips were flown, capturing 45,254 images with red, green, and blue (RGB) channels. The total surveyed area covered approximately 526.24 km^2^, with a data volume of 727 GB. The image set captured a wide variety of landforms, buildings, and a substantial population of livestock, including cattle, horses, and sheep, within the surveyed region.

**Figure 2:**
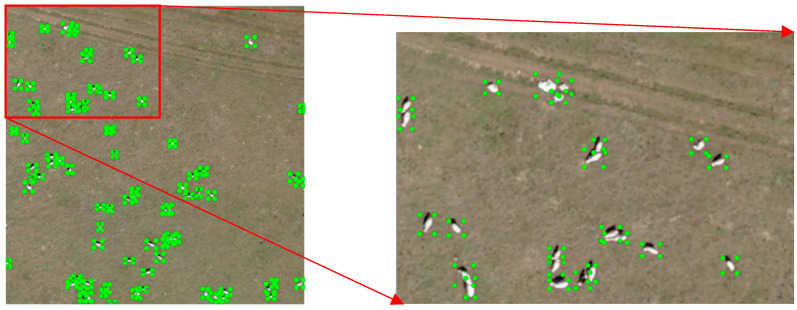
The annotation of samples.

**Figure 3:**
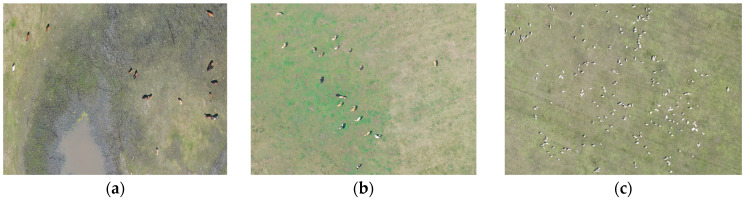
Dataset images: (**a**) herd of horses (**b**) herd of cattle (**c**) herd of sheep.

**Figure 4:**
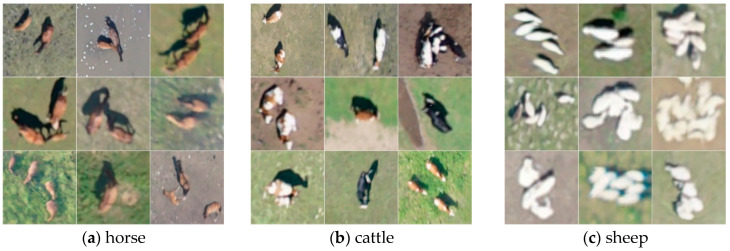
The object instances of the data.

**Figure 5:**
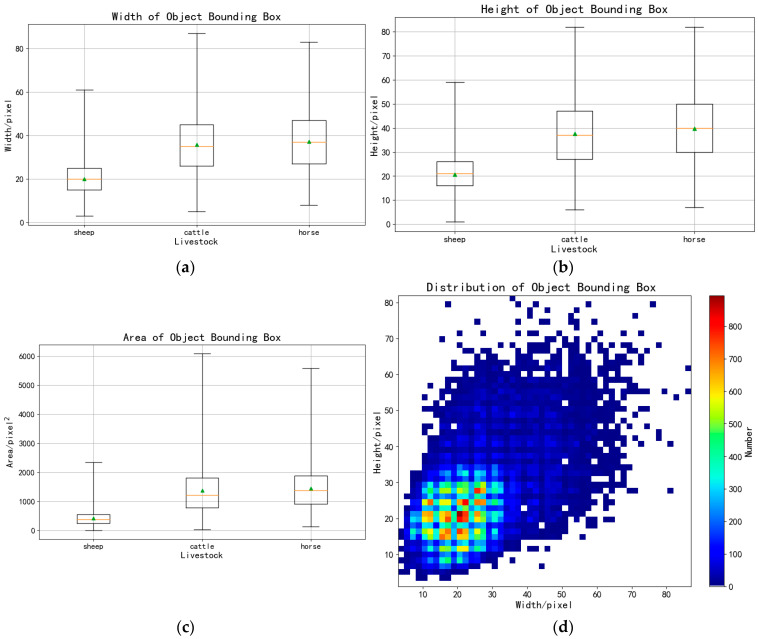
(**a**) The boxplot of object bounding box width. (**b**) The boxplot of object bounding box height. (**c**) The boxplot of object bounding box area. (**d**) The distribution of the object bounding boxes in the dataset. The horizontal axis is the width of the object bounding boxes and the vertical axis is the height. The color represents the number of boxes with the width and height in the coordinate system. The yellow horizontal line in the figure represents the median of the bounding box sizes for each type of livestock, which reflects the central tendency of the overall distribution. The green triangle indicates the mean, representing the average size across all samples.

In this study, the original UAV images (45,254 images), after being stitched using professional software, were annotated with polygonal boxes in ArcGIS, which were subsequently converted into rectangular boxes through a custom code. The UAV images obtained had a resolution of 7952 × 5304 pixels. To accommodate the limited memory of graphics cards, the original images were divided into subpatches of 1024 × 1024 pixels. Additionally, adjacent patches were designed to overlap, ensuring that any livestock instances split by cut-off lines would be fully detected in the overlapping areas. Based on this approach, the width of the overlapping region was set to 512 pixels. A total of 4396 subpatches were generated for dataset construction. The dataset was randomly partitioned into training, validation, and testing sets using common ratios: 9:1 for training and validation versus testing, and 9:1 for training versus validation, as shown in Table 1. Figure 2 displays sample images from the dataset, which includes three types of livestock: cattle, horses, and sheep. Figure 3 shows images of sheep flocks, cattle herds, and horse groups in the dataset. Figure 4 illustrates object instances of cattle, sheep, and horses within the dataset. Table 2 and Figure 5 provide the statistics for the detection boxes in the dataset. As indicated in Figure 5d, most object boxes have side lengths ranging from 10 to 20 pixels, which corresponds to the typical size range for sheep objects.

### 2.2. Research Methods

Currently, object detection techniques based on deep learning are generally classified into two-stage and one-stage algorithms. Notable examples of two-stage algorithms include Fast R-CNN, Faster R-CNN, and R-CNN. While two-stage algorithms tend to offer higher accuracy, they demand substantial computational resources and do not meet real-time performance requirements [18]. To address the challenge of balancing real-time performance with accuracy, Redmon et al. introduced the YOLO algorithm [19], a simple and efficient one-stage object detection framework.

**Figure 6:**
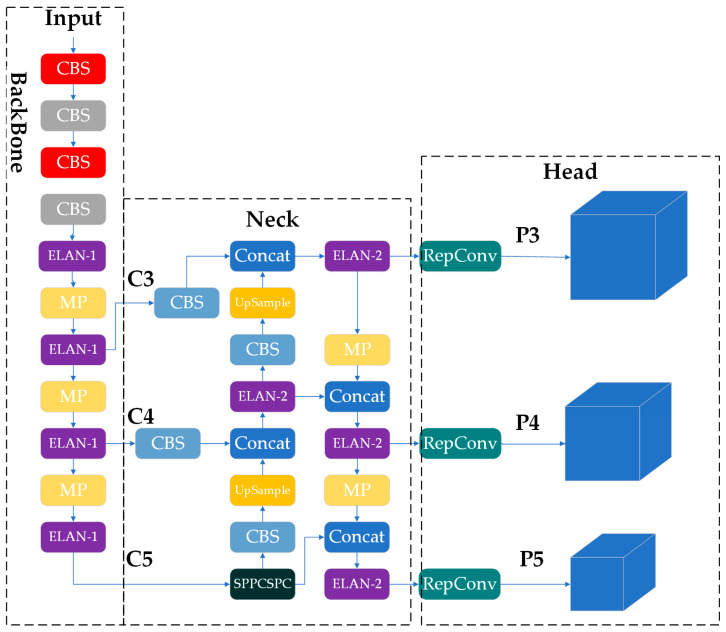
YOLOv7 network structure diagram.

**Figure 7:**
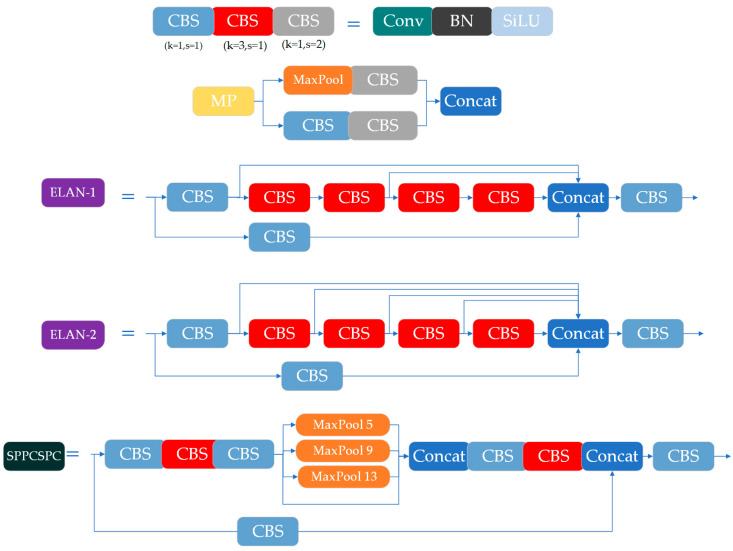
The modules of YOLOv7.

In their work on the YOLO algorithm, Wang et al. [20] proposed the YOLOv7 algorithm (Figure 6), whose overall architecture is depicted in Figure 6. YOLOv7 consists of three main components: the backbone, the neck, and the head. The backbone integrates Conv2D_BN_SiLu (CBS), ELAN, MP, and SPPCSPC modules. The CBS layer involves passing the image through a series of two-dimensional convolution layers, batch-normalization layers, and the SiLU activation function. The ELAN layer utilizes a multi-branch stacking process, with each branch containing a different number of CBS, resulting in a denser residual structure. This design facilitates network optimization and mitigates the gradient explosion problem commonly associated with deeper neural networks. The MP module, which performs down-sampling, differs from typical transition modules that rely on convolution or maximum pooling. In YOLOv7, Wang combined and stacked two transition modules to improve target-specific feature extraction. The SPPCSPC module extracts receptive fields at multiple scales via maximum pooling operations, which are then merged with previous feature maps, thereby enriching feature information through fusion. The structure of each of the above modules is shown in Figure 7.

The neck employs a path-aggregation network (PANet) structure to fuse feature layers at multiple scales, while the head incorporates the REP module to adjust the channel sizes of output features at various scales. These features are subsequently fed into the prediction head to finalize category predictions and anchor target bounding boxes. Therefore, the LSNET proposed in this paper represents an enhancement of the YOLOv7 framework.

#### 2.2.1. LSNET

**Figure 8:**
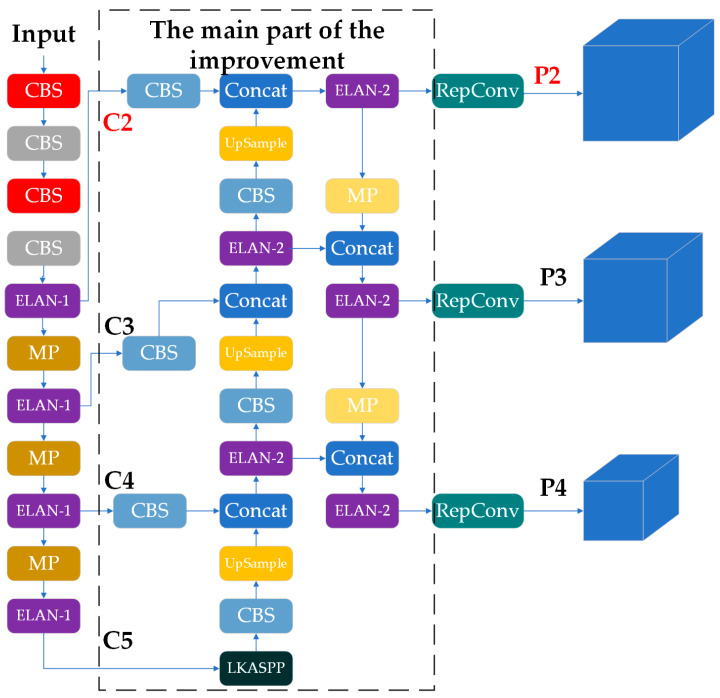
Network structure of the LSNET.

To systematically extract hierarchical features from input images, this study maintains the core components of the conventional YOLOv7 architecture, specifically retaining the E-ELAN and the MP for spatial feature transformation. The architectural modifications proceed through three sequential phases: First, an auxiliary P2 prediction head with corresponding network connections is incorporated into the framework, adopting identical structural configurations as the existing P3, P4, and P5 prediction heads to enhance shallow feature representation. Second, the original P5 prediction head and its associated network units are strategically removed to mitigate computational redundancy. Finally, a novel LKASPP module is developed, which integrates an inverted bottleneck structure with a large kernel attention mechanism to effectively capture discriminative high-level semantic features across multiple scales. The specific structure of LSNET is shown in Figure 8.

#### 2.2.2. Extra Prediction Head (P2)

In the complex background of UAV imagery, accurately detecting livestock is challenging due to their small size and dense distribution. To address this, an additional detection head, P2, is incorporated into the traditional YOLOv7 network to improve livestock detection in UAV images. The P2 prediction head is generated from the shallow, high-resolution feature map C2 [21]. Compared to other feature maps, C2, being a shallow feature map, retains more spatial information. The feature maps C2, C3, C4, and C5 are all extracted by the backbone network with down-sampling factors of 4, 8, 16, and 32, respectively. After multiple down-sampling operations, P5 has the largest anchor box size and receptive field. However, this process results in a significant loss of spatial information for small objects, leading to suboptimal feature fusion in the neck network and reduced detection accuracy [22]. In contrast, the prediction head P2, which corresponds to smaller anchor box sizes and receptive fields, allows the backbone network to capture finer-grained information. Therefore, to enhance the detection of small objects, the P2 prediction head replaces the P5 prediction head, and the network units containing the P5 detection head are removed. While the introduction of the additional P2 prediction head increases the number of parameters in the network, the removal of the P5 prediction head mitigates this increase.

An anchor-based object detector is sensitive to the size of the anchor box. Therefore, the K-means++ algorithm is employed to adjust the anchor box size for small objects. Table 3 shows the correspondence between the three prediction heads and the anchor box sizes for the three scales when the input image size is 640 × 640.

#### 2.2.3. WIoU v3 Loss Function

The IoU metric serves as the foundational formulation for bounding box regression loss in the original YOLOv7 framework [23]. Its subsequent evolution, CIoU, introduces static geometric parameterization through the following mathematical formulation:

$$L_{CIoU}=1-IoU+\frac{\rho^{2}(b,b_{g^{t}})}{c^{2}}+\alpha v$$

$$v=\frac{4}{\pi^{2}}{\left(arctan\frac{w^{g^{t}}}{h^{g^{t}}}-arctan\frac{w}{h}\right)}^{2}$$

$$\alpha =\frac{v}{\left(1-IoU\right)+v}$$

*b* and *b_gt_* represent the central points of the predicted bounding box and the ground-truth bounding box, respectively. *c* denotes the diagonal length of the smallest enclosing box covering both boxes, and ρ represents the distance between the central points of the two boxes. α is used to balance parameters, while v is used to evaluate the consistency of the aspect ratio.

While CIoU demonstrates empirical effectiveness in standard detection benchmarks with high-quality imagery, critical limitations emerge when deployed in challenging real-world scenarios [24]. First, the static parameterization scheme lacks explicit mechanisms to differentiate between high-quality and low-quality training samples, potentially compromising model generalizability under long-tailed data distributions. Second, the aspect ratio penalty term imposes artificial constraints that poorly align with the natural variance of object proportions in practical detection environments, particularly when handling non-axis-aligned targets. These limitations become particularly pronounced in UAV-based livestock monitoring applications characterized by: Low-resolution targets with ambiguous boundaries, Frequent inter-object occlusions within dense herds, and Inherent noise in aerial training and notations.

Under these conditions, the geometric constraints of CIoU indiscriminately amplify penalty terms for ambiguous samples, disproportionately penalizing predictions involving partial occlusions or boundary uncertainties. This systematic bias ultimately undermines model robustness by overfitting to idealized annotation assumptions, rather than adapting to the complexities of real-world detection. To address these challenges, our work proposes dynamic loss formulations that automatically adjust geometric constraints based on the detection difficulty of each sample.

**Figure 9:**
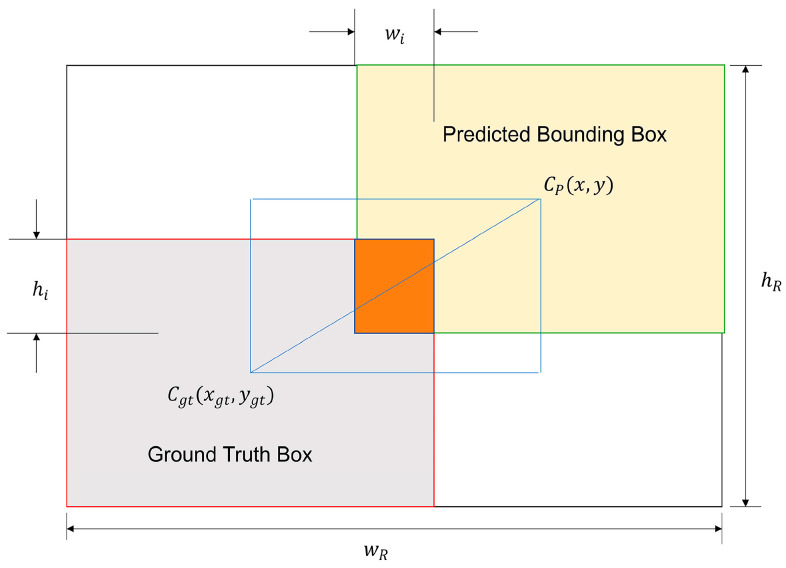
The schematic diagram of WIoU parameters.

To address the limitations of conventional bounding box regression approaches, the Wise-IoU (WIoU) framework [25] was developed, incorporating a dynamic non-monotonic gradient modulation mechanism. As schematically depicted in Figure 9, this advanced loss formulation comprises three progressive iterations: WIoU v1, v2, and v3, each introducing distinct geometric constraint strategies. The foundational WIoU v1 implementation establishes spatial distance as a critical attention metric through its dual-layer adaptive weighting architecture, mathematically expressed as:

$$L_{WIoUv1}=R_{WIoU}*L_{IoU}$$

$$R_{WIoU}=\exp(\frac{{\left(b_{c_{x}}^{g^{t}}-b_{c_{x}}\right)}^{2}+{\left(b_{c_{y}}^{g^{t}}-b_{c_{y}}\right)}^{2}}{\left(c_{w^{2}}+c_{h^{2}}\right)})$$

$$L_{IoU}=1-IoU$$

For the predicted bounding box, $b_{c_{x}}$ and $b_{c_{y}}$, respectively, represent the center coordinates. Similarly, $b_{c_{x}}^{g^{t}}$ and $b_{c_{y}}^{g^{t}}$ describes the center coordinates of the ground-truth bounding box.

WIoU v2 incorporates a monotonic focusing mechanism specifically tailored for cross-entropy loss. Building upon WIoU v1, WIoU v3 defines an outlier parameter β to quantify the quality of anchor boxes and constructs a non-monotonic focus factor r based on β.

$$\beta =\frac{{L^{*}}_{IoU}}{L_{IoU}}\in [0,+\infty ]$$

A smaller outlierness β implies a higher quality anchor box, resulting in a smaller gradient boost assigned to it, allowing for better bounding box regression focus on anchor boxes with common quality.

$$L_{WIoUv3}=r*L_{WIoUv1},\;r=\frac{\beta }{\delta \alpha^{\beta -\delta }}$$

where *δ* makes *r* = 1 when *β* = *δ*. The anchor box will enjoy the highest gradient gain when its outlier degree satisfies *β* = C (C is a constant value).

Through the comparative analysis mentioned above, this study achieved a significant improvement by replacing the traditional CIoU with WIoU v3 in YOLOv7. WIoU v3 weighs the learning of low-quality examples and high-quality examples., which makes the model focus more on anchor boxes of ordinary quality and improves the model’s ability to localize objects. For the livestock object detection task in the UAV aerial photography scene, WIoU v3 dynamically allocates gradient boosts according to the current situation at any given moment.

#### 2.2.4. LKASPP (Large Kernel Attentions Spatial Pyramid Pooling)

**Figure 10:**
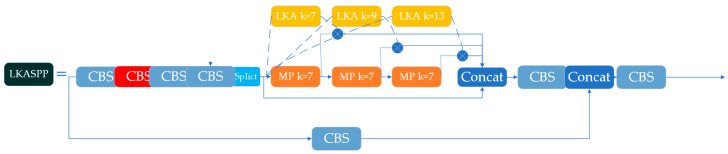
LKASPP.

**Figure 11:**
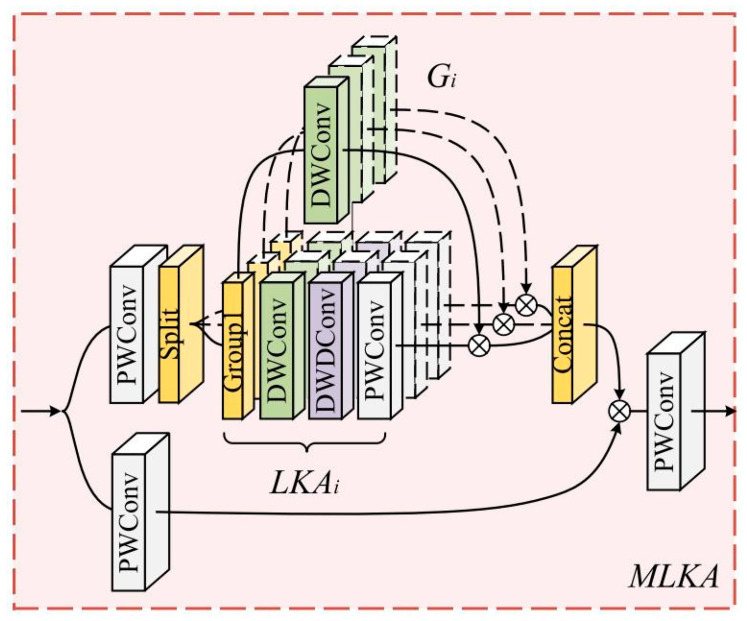
MLKA.

SPPCSPC achieves effective multi-scale feature extraction and fusion by incorporating three parallel pooling layers (kernel sizes 5, 9, 13) within a convolutional series and integrating them with the CSPN (Cross Stage Partial Network) structure. The CSPN reduces computational load by splitting feature maps for reuse while preserving the model’s representational capacity. However, the 13 × 13 max pooling layer in SPPCSPC entails substantial computational overhead. To mitigate this, LKASPP (Figure 10) adopts three sequential 7 × 7 max pooling layers, enabling the fused structure to attain an expanded receptive field. While max pooling enlarges the receptive field, it may induce feature degradation. Furthermore, parallel pooling layers fail to simultaneously capture local information and model long-range dependencies. To address these limitations, we integrate Multi-scale Large Kernel Attention (MLKA) [26] into the module. MLKA (Figure 11) comprises three key components: (1) large kernel attention (LKA) to establish channel interdependence, (2) a multi-scale mechanism to capture hierarchical correlations, and (3) gated aggregation for dynamic recalibration. The LKA adaptively establishes long-range relationships by decomposing a convolution into three sequential operations: a depth-wise convolution, a depth-wise dilated convolution, and a point-wise convolution. This hybrid design leverages depth-wise dilated convolutions to expand the receptive field while maintaining computational efficiency, coupled with point-wise convolutions to fuse cross-channel features. The improved model employs sequential 7 × 7 max pooling operations to capture local context, superseding the original spatial gating mechanism. Through this adaptation, the redesigned MLKA dynamically adjusts the LKA, enabling the model to jointly prioritize fine-grained details and global structural cues. Consequently, the LKASPP framework significantly enhances high-level semantic feature extraction capabilities).

## 3. Results

In this experiment, the algorithm was executed on a CP12 vCPU Intel(R) Xeon(R) Silver 8362 CPU @ 2.80 GHz, with 45 GB of memory, an RTX 3090 GPU, CUDA 11.3, Python 3.8, and PyTorch 1.11.0. The model was trained for 300 epochs using the SGD optimizer, with momentum set to 0.937 and weight decay set to 4 × 10^−5^. The learning rate was initially set to 0.01, with a minimum value of 0.0001. The cosine annealing method was employed for learning rate decay.

To validate the effectiveness of our proposed components, we conducted ablation studies on the livestock dataset. The results are presented in Table 4. The first row shows the results for the standard YOLOv7, while the subsequent rows display the outcomes for slightly modified versions. As indicated in the second row of Table 4, removing the P5 prediction head resulted in a slight reduction in performance (−0.07% mAP compared to YOLOv7). This outcome demonstrates that the removal of the P5 prediction head has a negligible impact on the detection of small livestock targets. The results in rows 2, 3, and 4 further illustrate the effect of incorporating the P2 prediction head. Replacing the P5 layer with the P2 layer led to improved performance in detecting small objects. Furthermore, the GFLOPS only increased by 7.456 G, while the number of parameters decreased by 11.193 M. As shown in the fifth row of Table 4, when the LKASPP module was added, the performance increased from 92.40% to 93.10%. The sixth row presents a notable improvement in performance, with an increase from 93.10% to 93.33%. This increase provides compelling evidence of the efficacy of the WIoU v3 loss function in small target detection.

**Figure 12:**
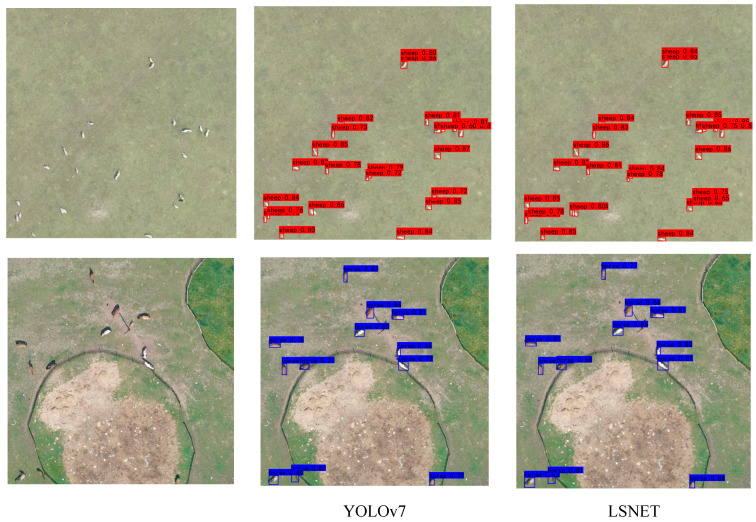
The result of the ablation experiments.

As shown in Table 4, the proposed LSNET algorithm, composed of the above methods, achieved a 1.47% mAP@0.5 improvement compared to the traditional YOLOv7. Although the GFLOPS increased by 6.9713 G, the number of parameters was reduced by 11.193 million, which is approximately one-third of the original model’s parameters. Some detection results on the Hulunbuir livestock dataset are shown in Figure 12. The bounding boxes of different categories are labeled with category names and corresponding confidence scores. The results show that the proposed LSNET algorithm is more effective in detecting small objects than the original model.

**Figure 13:**
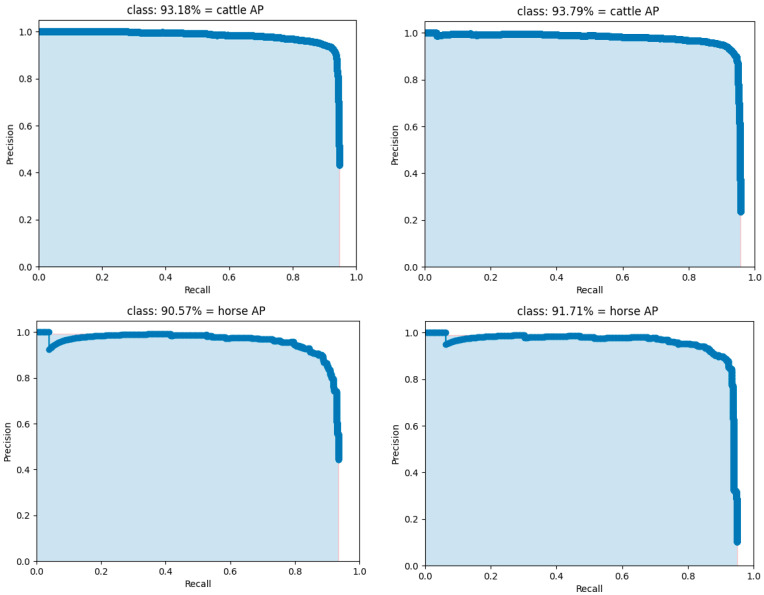

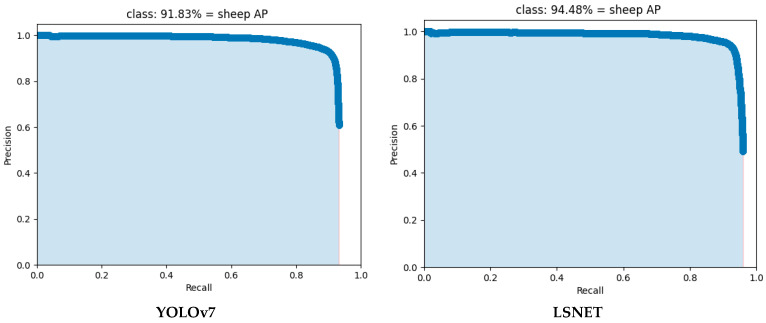
PR curve.

**Figure 14:**
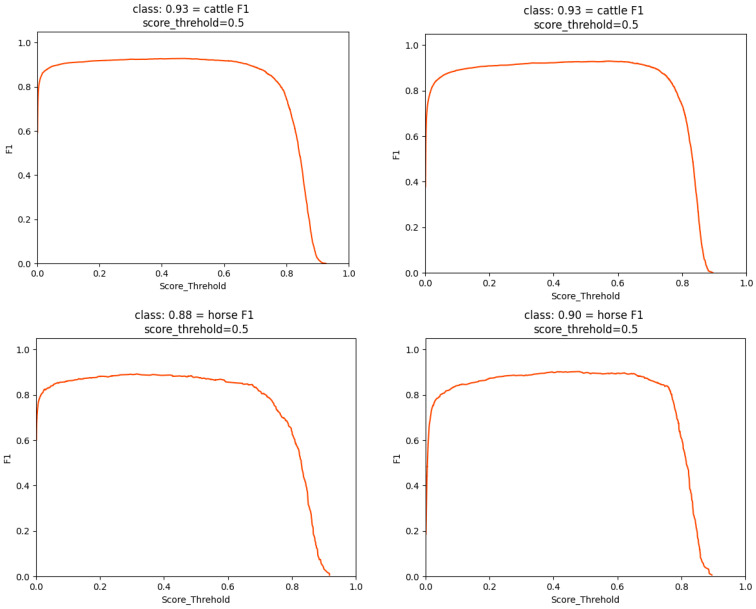

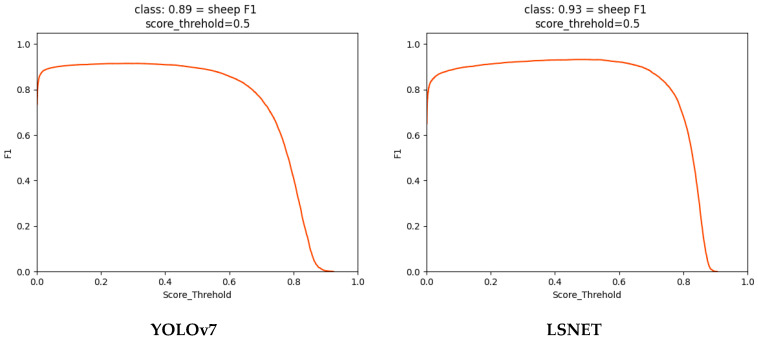
F1 Score Comparison Plot.

As shown in Figure 13, the PR comparison between YOLOv7 and LSNet demonstrates that the area under the PR curve of LSNet for detecting cattle, sheep, and horses significantly outperforms that of YOLOv7. From the perspective of Precision, LSNet maintains a higher Precision at the same Recall compared to YOLOv7, particularly in the high Recall range, where the decline in Precision is more gradual. This indicates that LSNet has fewer false positives (FP), meaning it misidentifies fewer non-livestock objects. From the Recall perspective, LSNet achieves a higher Recall at the same Precision compared to YOLOv7, indicating a lower false negative (FN) rate. This suggests that LSNet is capable of detecting more livestock targets with fewer missed detections, which is particularly evident in the sheep category. Specifically, LSNET achieves AP improvements of 0.61%, 1.16%, and 2.65% over YOLOv7 for these three categories, respectively. As shown in Figure 14, LSNET generally outperforms YOLOv7 in terms of scores across all classes, particularly at higher thresholds, where LSNET maintains a higher F1 score for the cattle and horse classes. Specifically, LSNET excels in balancing precision and recall, especially in applications that require high-precision predictions. Compared to YOLOv7, LSNET is more stable in detecting specific classes and is better at maintaining high precision at higher thresholds.

## 4. Discussion and Potential Future Works

### 4.1. Discussion

To demonstrate the effectiveness and advantages of the proposed method, LSNET was compared with various deep learning-based object detection models, such as Faster R-CNN [27], CenterNet [28], YOLOv5 and other models from the YOLOv7 series. These models encompass single-stage, two-stage, anchor-based, and anchor-free architectures. The performance of these models on our dataset is detailed in Table 5. LSNET achieves improvements of 73.72%, 39.52%, 3.48% and 1.78% over Faster R-CNN, CenterNet, YOLOv5 and YOLOv7-X.

**Figure 15:**
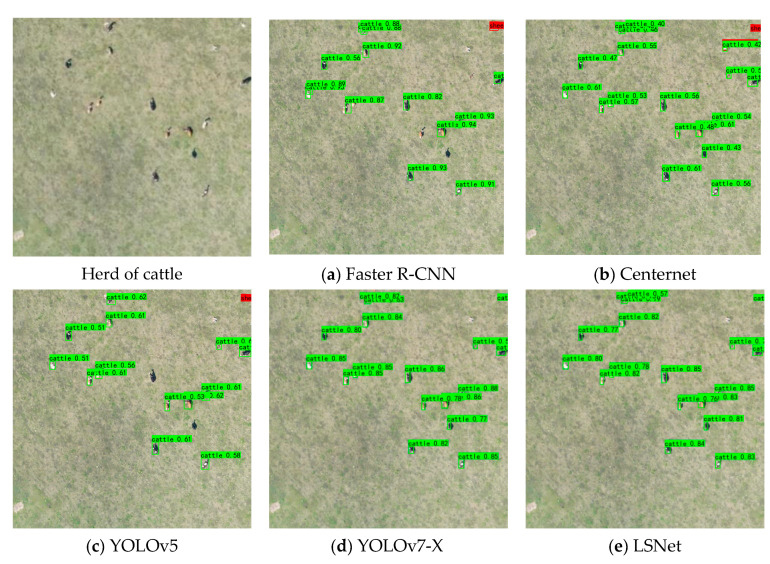
Detection results for cattle.

**Figure 16:**
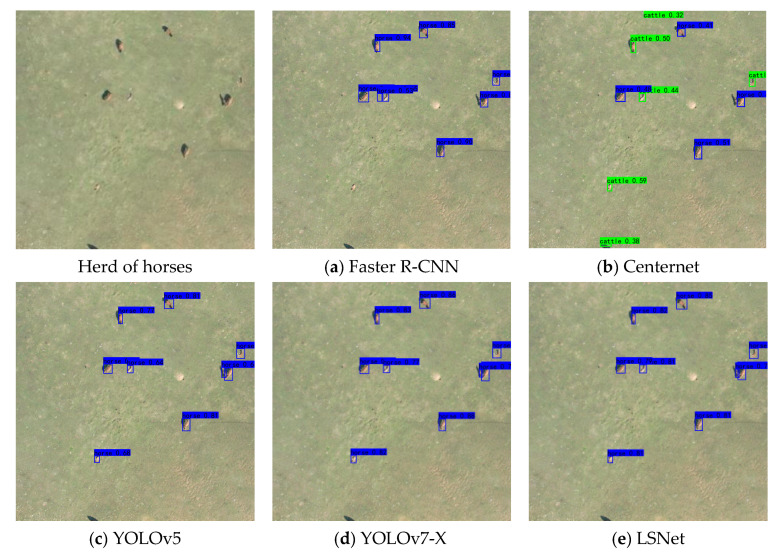
Detection results for horses.

**Figure 17:**
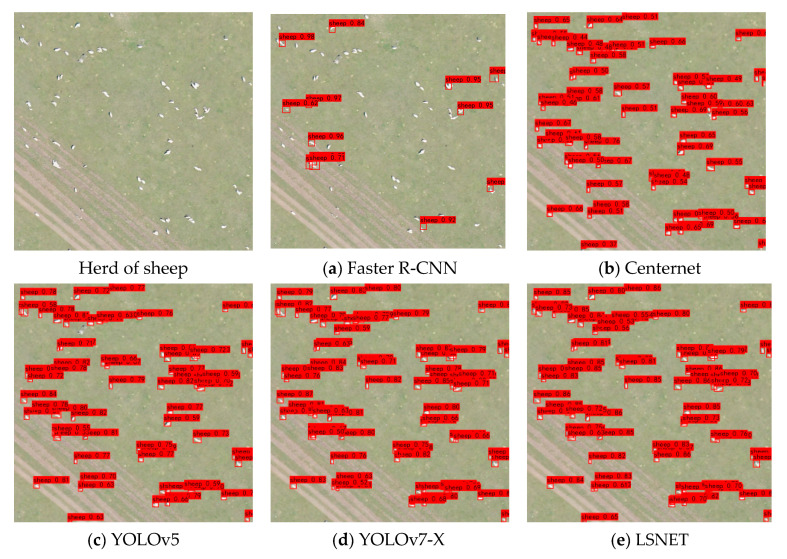
Detection results for sheep.

Visual comparisons of different methods are presented in Figure 15, Figure 16 and Figure 17. Among the approaches evaluated, two-stage algorithms like Faster R-CNN exhibit slower detection speeds compared to their one-stage counterparts. Additionally, Faster R-CNN is an anchor-based model, and its accuracy is highly dependent on the proper setting of anchor hyperparameters. Despite a series of basic hyperparameter adjustments, the experimental results yielded only a 19.61% mAP, with numerous missed and incorrect detections. CenterNet, an anchor-free object detection algorithm introduced by Duan et al., eliminates the need for anchors; however, it struggles with small-object occlusion and dense scenes, leading to challenges in distinguishing between cows and horses. YOLOv5, while demonstrating relatively high detection accuracy, still faces issues with missed detections for sheep.

Among the YOLOv7 variants, the YOLOv7-X model, with the highest number of parameters in the YOLOv7 series, achieved an mAP of 92.09%. In comparison, the proposed LSNET model reached an mAP of 93.33%, slightly outperforming YOLOv7-X. Notably, the LSNET model has only 26.247 million parameters, approximately 50% of the number of parameters in YOLOv7-X.

LSNET demonstrated high detection accuracy, with no instances of missed detections or false positives. The visual and quantitative comparisons highlight the efficacy of the proposed LSNET in livestock detection tasks.

### 4.2. Potential Future Works

The possibility of conducting large-scale livestock surveys in Hulunbuir is now more feasible than ever, particularly with the use of fixed-wing UAVs. These UAVs allow for the coverage of significantly larger areas with high-resolution imagery in a single flight, offering a clear advantage over unmanned gyroplanes. When coupled with computer vision models, livestock surveys can become a valuable tool for ecosystem monitoring. This study introduces the LSNET model for livestock detection, which can be deployed for surveys in various regions without the need for human intervention. Experimental results demonstrate that the proposed LSNET outperforms baseline models, effectively addressing the challenges of detecting small livestock while minimizing false positives and missed detections. Furthermore, while the focus of this work is livestock detection, the model can be extended to more generalized automated animal species detection, making it suitable for comprehensive and systematic wildlife surveys.

However, there are several areas for improvement. First, the computational efficiency of the model is not yet fully optimized. Second, the model struggles with detecting ultra-dense livestock populations. In addition to these challenges, there are other promising avenues for future research. Currently, the model is primarily applicable to UAV images captured under clear weather conditions, and its practical performance has not been thoroughly evaluated in adverse atmospheric conditions, such as fog or rain. To enhance the model’s robustness and generalization, it is essential to collect more diverse image datasets that include varying livestock types, environmental conditions, and lighting scenarios. Furthermore, investigating domain adaptation techniques, such as transfer learning, could enable the model to better adapt to different environments or regions, improving its cross-domain applicability. Despite reducing the parameter count to 26.247 million, the optimized model still faces computational constraints that make it unsuitable for deployment on UAVs. To address this limitation, future work could explore techniques like model pruning and quantization, which would reduce computational and storage requirements, enabling more efficient deployment on UAVs. In addition, LSNET can be deployed on a server, and by using a mobile app to call the server’s API, it is possible to retrieve UAV image detection results on the mobile phone.

## 5. Conclusions

In response to the challenges posed by small, densely distributed livestock targets in aerial remote sensing images—targets that are easily obscured by background noise—this study introduces a specialized small-target livestock dataset based on UAV imagery. We propose the LSNET algorithm, an efficient model designed to enhance livestock detection in UAV images, with the goal of better integrating deep learning techniques into remote sensing applications for grassland animal husbandry. The model’s ability to detect small, easily confused livestock targets is strengthened through the incorporation of the P2 prediction head, the WIoU v3 loss function, and the LKASPP module. Experimental results demonstrate that LSNET achieves an impressive mean mAP of 93.33% on the Hulunbuir livestock dataset, showcasing its high applicability to UAV imagery.

The insights gained from this model offer significant potential for monitoring livestock distribution and density across vast grassland ecosystems—data critical for both ecological studies and sustainable grazing practices. By providing a comprehensive view of livestock populations, the model aids in better management and monitoring, while contributing to the preservation of grassland ecosystems. Overall, the LSNET algorithm proves highly effective in addressing the major challenges of livestock detection and enables large-scale livestock surveys in expansive grassland areas.

## Figures and Tables

**Table 1:** The allocation of images.

Datasets	Animal Patches
Training	3560
Validation	396
Testing	440
Total	4396

**Table 2:** Details of objects’ sizes.

Category	Min Width	Max Width	Average Width	Min Height	Max Height	Average Height	Average Area	Number
cattle	5.0	87.0	35.71	6.0	82.0	37.67	1453.64	20,486
sheep	3.0	61.0	20.04	1.0	59.0	20.89	424.73	74,753
horse	8.0	83.0	37.16	7.0	82.0	39.71	1453.65	2788

**Table 3:** Anchor box size setting in LSNET.

Prediction Head	Feature Graph	Anchor Size
P2	160 × 160	(11, 6) (8, 9) (7.5, 14)
P3	80 × 80	(16, 8) (15, 11) (12,14)
P4	40 × 40	(18, 16) (13, 22) (28, 27)

**Table 4:** Ablation study on the Hulunbuir grassland grazing livestock dataset.

Model	Category	AP@0.5 (%)	F1	Recall (%)	Precision (%)	mAP (%)	Parameters (M)	GFLOPS (G)
YOLOv7	cattle	93.18	0.93	91.43	93.72	91.86	37.620	106.472
	horse	90.57	0.88	83.12	93.09			
	sheep	91.83	0.89	83.87	95.79			
YOLOv7 + remove YOLOv7-P5	cattle	93.53	0.93	91.81	94.39	91.79	26.338	98.120
	horse	90.20	0.89	82.14	92.67			
	sheep	91.65	0.90	84.21	95.94			
YOLOv7 + P2	cattle	93.23	0.93	93.91	92.60	92.12	38.20	123.122
	horse	89.33	0.89	88.38	89.96			
	sheep	93.80	0.93	90.49	94.70			
YOLOv7 + remove YOLOv7-P5 + P2	cattle	93.89	0.93	93.12	92.78	92.40	26.920	113.928
	horse	88.72	0.89	85.71	92.96			
	sheep	94.60	0.93	91.39	95.06			
YOLOv7 + remove YOLOv7-P5 + P2 + LKASPP	cattle	93.72	0.93	93.25	92.12	93.10	26.247	113.385
	horse	91.33	0.90	88.37	90.78			
	sheep	94.24	0.93	9.34	95.03			
YOLOv7 + remove YOLOv7-P5 + P2 + LKASPP +WIoU v3(LSNET)	cattle	93.79	0.93	92.79	92.51	93.33	26.247	113.385
	horse	91.71	0.90	89.37	90.27			
	sheep	94.48	0.93	91.75	94.59			

**Table 5:** Comparison of state-of-the-art object-detection models.

Model	Category	AP@0.5 (%)	F1	Recall (%)	Precision (%)	mAP (%)	Parameters (M)
Faster R-CNN	cattle	32.97	0.45	53.38	38.99	19.61	28.3
	horse	23.59	0.38	49.80	30.49		
	sheep	2.28	0.09	5.17	30.70		
Centernet	cattle	64.17	0.50	34.68	89.43	53.38	32.665
	horse	44.82	0.13	74.17	85.19		
	sheep	48.86	0.56	43.13	81.36		
YOLOv5	cattle	92.65	0.92	90.61	93.23	89.89	47.057
	horse	88.01	0.88	85.27	90.22		
	sheep	89.02	0.88	82.10	95.15		
YOLOv7_X	cattle	93.31	0.93	91.96	93.94	92.09	71.344
	horse	90.37	0.89	88.31	90.67		
	sheep	92.58	0.91	86.20	92.09		
LSNET	cattle	93.79	0.93	92.79	92.51	93.33	26.247
	horse	91.71	0.90	89.37	90.27		
	sheep	94.48	0.93	91.75	94.59		

## Data Availability

The data are not publicly available due to their containing information that could compromise the privacy of research participants.
